# Age estimation of children based on open apex measurement in the developing permanent dentition: an Egyptian formula

**DOI:** 10.1007/s00784-022-04773-7

**Published:** 2022-11-17

**Authors:** Shaimaa S. El-Desouky, Ibrahim A. Kabbash

**Affiliations:** 1 Pediatric Dentistry, Oral Health and Preventive Dentistry Department, Faculty of Dentistry, Tanta University, Tanta, Egypt; 2 Public Health & Community Medicine Department, Faculty of Medicine, Tanta University, Tanta, Egypt

**Keywords:** Forensic dentistry, Dental age, Chronological age, Cameriere method

## Abstract

**Background:**

Cameriere’s original formula based on open apex measurements is a reliable, clinically applicable method for dental age estimation in different populations children. Dental development may differ between Egyptian children and other ethnic populations which may affect dental age accuracy using Cameriere’s formula.

**Aim:**

Firstly, to verify Cameriere’s original formula on large Egyptian children sample, secondly, to develop an Egyptian-specific formula based on Cameriere’s method.

**Material and methods:**

A prospective cross-sectional study of 762 good quality Orthopantomograms (OPGs) of 5–15 aged healthy Egyptian children selected from Nile Delta governorates between August 2020 and December 2021. Chronological age (CA) was calculated by subtracting birth date from radiograph date. OPGs were analyzed for ***N***_**0**_, ***S***, ***X***_**i**_ morphologic variables using Sidexis program after that dental age was calculated using Cameriere’s formula then compared to CA. Multiple linear regression model was used to adapt Cameriere’s formula to construct an Egyptian formula. The same sample was used to verify the new formula accuracy.

**Results:**

A total of 1093 OPGs were collected; 762 OPGs which met inclusion criteria were analyzed. Cameriere’s original formula revealed − 0.59- and − 0.53-year underestimation of females and males dental age (DA) respectively (*p* < 0.001). Regression analysis using the morphologic variables showed that *X*_4_, *X*_7_, *N*_0_ contributed significantly to CA yielding Egyptian-specific formula. New formula showed − 0.12-year male underestimation and 0.1-year female overestimation (*p* > 0.05).

**Conclusion:**

Egyptian formula was more accurate than Cameriere’s formula in Egyptian children.

**Clinical relevance:**

Egyptian-specific formula decreases the gap between CA and DA, so a relative approximate age is obtained that helps proper diagnosis and treatment planning for orthodontic and pediatric dentistry problems.

## Introduction

Forensic dentistry is one of the most fascinating and unexplored branches of forensic science [1]. It is primarily concerned with identification based on the recognition of distinctive characteristics present in an individual’s dental structures [2]. Age estimation is extremely crucial for identifying deceased victims of crimes, disasters, and accidents [3]. Age calculation is useful in situations such as jobs, marriage, felons, legalization, and immigration approval [4]. In the dental field, age estimation is essential for accurate diagnosis and treatment planning for orthodontic and pediatric patients [5]. Dental maturation is considered to be independent than somatic, skeletal, and sexual maturation because teeth are derived from different embryonic origins, and thus, under different control mechanisms, the former being mesodermal in origin while dental structure is derived from ectomesenchyme [6]. When compared to other maturity indicators [7–10], dental maturity indicators have been proposed as more validated and accurate parameters for age estimation in children and adolescents.

Several methods for estimating age have been developed depending on radiographic examination of the degree of tooth development [11–16]. The eight-stage system proposed by Demirjian [17] is one of the most widely used methods for calculating dental age. This system determines age by assessing the developmental stages of the left mandibular permanent teeth using panoramic radiographs. However, a significant number of research have reported that the French–Canadian standards introduced by Demirjian were not suitable for age estimations of children in other regions of the world, with an overall tendency of age overestimation compared to chronological age [7, 15, 18–22].

The popular methods of assessing dental age have been criticized because they depend on subjective estimations of tooth development as seen in radiographs, followed by comparisons with illustrations and explanations in documented dental charts [23]. Objective methods of age calculation from developing teeth [23–27] were correlated with subjects’ chronologic ages in an attempt to avoid subjective estimations. Crown height, apex width, and root length were measured, and a linear correlation between some of these distances and age was formed using multiple regression equations. Mörnstad et al. [23] developed the first approach employing linear parameters from panoramic radiographs to build multiple regression models for dental age estimation in 541 Swedish children aged 6–14 years. On all 16 permanent mandibular teeth, seven molar tooth measurements and three premolar and single-rooted tooth measurements were taken. The claimed accuracy was startling, with predicted age being within several hours of actual age, which seemed impossible.

Cameriere et al. [27] introduced a linear regression formula for estimating dental age, based primarily on measurements of open apices in 455 Italian children aged 5 to 15 years. This method was evaluated on a large number of children from multiple European countries, including Croatia, Germany, Kosovo, Italy, Slovenia, Spain, and the United Kingdom, yielding a common formula effective for all these countries [28]. The validity and accuracy of Cameriere’s method were tested on several sample groups from various nationalities [29–33], revealing that the original regression model developed by Cameriere may not always be applicable to other countries because tooth development differed among populations and varied across ethnic groups and geographic areas[34]. Moreover, diet, socioeconomic status, nutritional habits, and lifestyle all have an impact on tooth development [19]. As a result, a few authors adjusted Cameriere’s regression model with newer samples and proposed a new formula to suit their populations [34–42].

In a comparative study by El-Bakary et al. [43], the applicability of Cameriere’s original formula and Willems’ methods were evaluated for an Egyptian sample (134 boys, 152 girls) with age range from 5 to 16 years. It was concluded that Cameriere’s formula underestimated the age and was less accurate than Willems’ method in Egyptian children [15]. Moreover, no studies have been conducted on large sample of the Egyptian children and adolescents to assess the accuracy of Cameriere’s original formula and to find out whether this formula is suitable for the Egyptian population or not, so the aims of present study are as follows: firstly, to verify Cameriere’s original age estimation model on a large sample of Egyptian children, and, secondly, to develop an Egyptian-specific formula based on Cameriere’s method.

## Material and methods

### Study design and setting

**Fig. 1:**
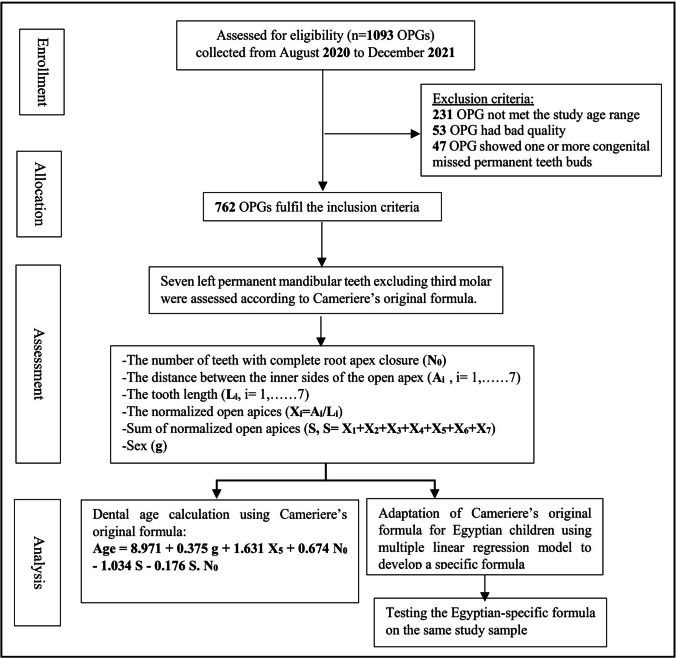
Flow chart of the present study showing sampling process

This prospective cross-sectional study was conducted in Nile Delta governorates (Gharbia, Menoufia, Dakahlia), Egypt between August 2020 and December 2021. A sample of 1093 OPGs was collected for healthy Egyptian children and adolescents aged 5–15 years who were in need for panoramic radiographs in their dental treatment. These OPGs were assessed according to study inclusion and exclusion criteria. The inclusion criteria were good quality radiographs and no agenesis or extractions in the left lower quadrant [40]. Children with premature birth, facial asymmetry, congenital anomalies, previously orthodontic treatment, history of trauma or surgery in dento-facial region, and hypodontia of permanent teeth except third molars or hyperdontia were excluded [41]. Flow chart including enrollment, allocation, assessment, and analysis of sample size is presented in Fig. 1. Accordingly, 231 OPGs were excluded as it was out of the study age range; also, 53 OPGs had bad quality and 47 OPGs showed one or more congenital missed permanent teeth buds. The final study sample was consisted of 762 OPGs of Egyptian children and adolescents aged 5–15 years (354 males and 408 females) (Table 1).

**Table 1:** Age and sex distribution of the studied patients

Age in years	Males		Females		Total	
	*n*	%	*n*	%	*N*	%
5	28	7.9	34	8.3	62	8.1
6	23	6.5	38	9.3	61	8.0
7	54	15.3	39	9.6	93	12.2
8	49	13.8	53	13.0	102	13.4
9	35	9.9	45	11.0	80	10.5
10	49	13.8	50	12.3	99	13.0
11	47	13.3	37	9.1	84	11.0
12	27	7.6	49	12.0	76	10.0
13	25	7.1	31	7.6	56	7.3
14	15	4.2	32	7.8	47	6.2
15	2	0.6	0	0.0	2	0.3
Total	354	100	408	100	762	100

### Sample size calculation

The sample size and power analysis were calculated using Epi-Info software statistical package created by World Health organization and center for Disease Control and Prevention, Atlanta, Georgia, USA, version 2002. The criteria used for sample size calculation were as follows: study design is a comparative cross sectional, 95% confidence limit. The sensitivity of age identification is 80% with a margin of error of 3%. The sample size based on the previously mentioned criteria was found at *N* > 682.

### Cameriere’s dental age versus chronological age

**Fig. 2:**
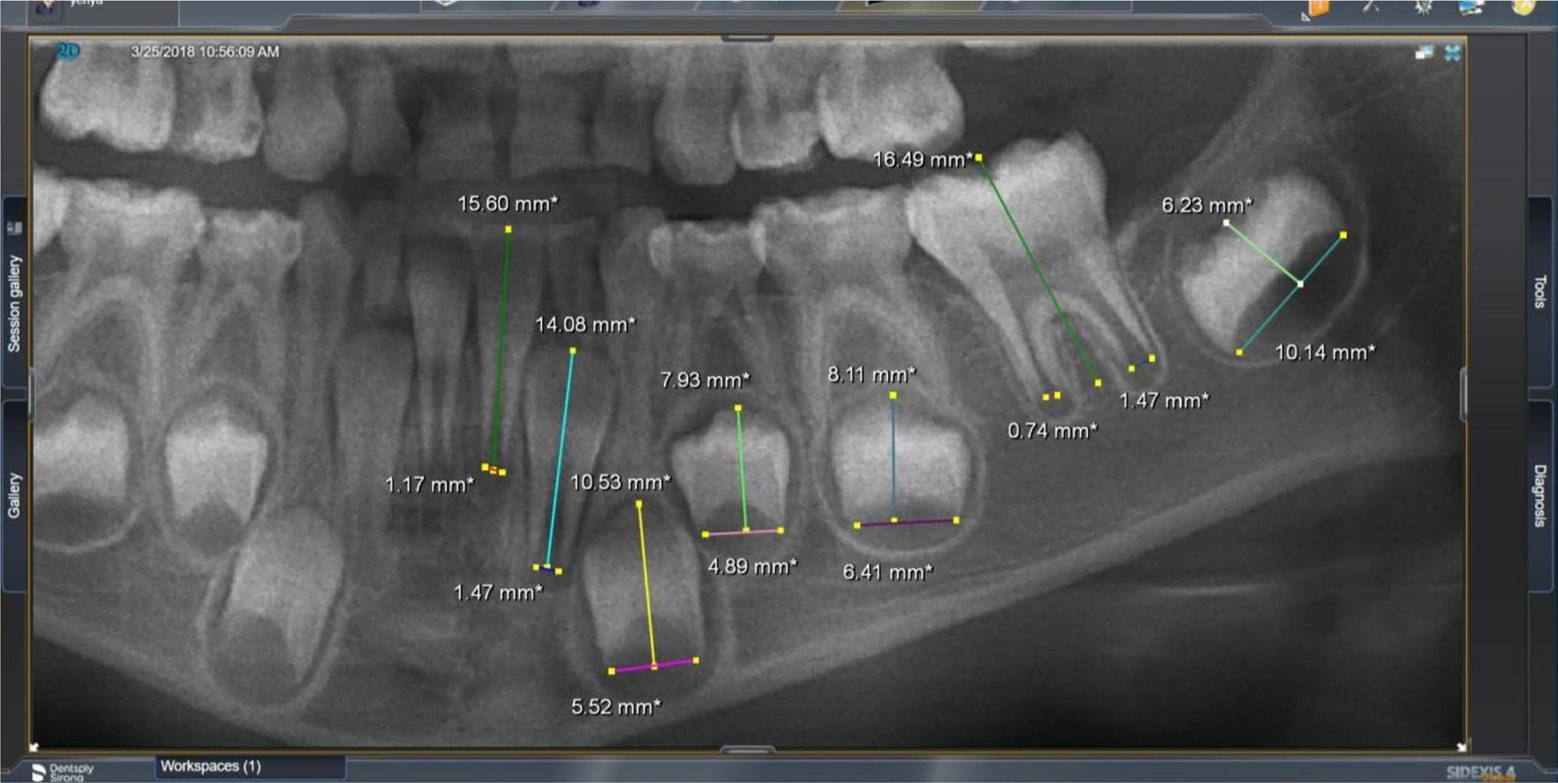
An example from our study group: Sidexis program analyzed a panoramic radiograph of 7.15 years male patient

Firstly, the personal data of patients especially sex, date of birth, and date of OPG was recorded. The chronological age (CA) was calculated by subtracting date of birth from date of radiograph and the result was converted to a decimal age. Patients were subjected to panoramic radiograph (Orthophos XG DS/ceph, Sirona); then, the panoramic images were processed using radiographic analysis program (Sidexis 4, ver 2.4.0.60310®, SIRONA Dental Systems GmbH). The seven left permanent mandibular teeth excluding third molar were valued (Fig. 2). The number of teeth with complete root apex closure was calculated as ***N***_**0**_. The teeth with open apices had been considered: For teeth with one root, the distance between the inner sides of the open apex was measured (***A***_**i**_) while the teeth with two roots, the sum of the distances between the inner sides of the two open apices was evaluated. The measurements had been normalized by dividing ***A***_**i**_ by the tooth length (***L***_**i**_). Finally, dental age had been calculated using the following Cameriere’s original formula [27]:

1 $$\mathrm{Age}=8.971+0.375\mathrm{ g}+1.631 {\mathrm{X}}_{5}+0.674 {N}_{0}-1.034\mathrm{ S}-0.176 \mathrm{S}. {N}_{0 }$$

where ***g*** is a variable equal to one for boys and zero for girls, ***X***_**5**_ is the normalized measurement of left mandibular second premolar which equal ***A***_**5**_**/*L***_**5**_, ***N***_**0**_ is the number of teeth with complete root apex closure, and ***S*** is the sum of the normalized open apices which equal ***X***_**1**_** + *X***_**2**_** + *X***_**3**_** + *X***_**4**_** + *X***_**5**_** + *X***_**6**_** + *X***_**7**_.

### Statistical analysis

Data were collected, tabulated, and analyzed using SPSS version 19 (Statistical Package for Social Studies) created by IBM, Illinois, Chicago, USA. For numerical values the range, mean and standard deviations were calculated. Correlation between dental and chronological age was calculated using Pearson’s correlation coefficient (*r*). All measurements were performed by the same examiner (first author); to verify the intra examiner reliability, 10% of all cases were assessed twice after a wash out period of 1 month by the researcher and Kappa test [44] was calculated. The differences between mean value of chronological age and Cameriere dental age at each age category were tested by paired *t*-test, a positive result indicates overestimation while a negative result indicates underestimation. The level of agreement between chronological age and dental age was tested using kappa test [44]. The level of significance was adopted at *p* < 0.05.

### New formula construction

For each of the analyzed OPGs, the morphologic variables, ***N***_**0**_, ***X***_**i**_, where ***i*** = tooth 1…7, and the subject’s gender were used as predictive variables for age estimation in sequential statistical analysis. To simplify the Egyptian equation, the variable “*S*” was discarded since it is dependent on the normalized measurement of all left lower seven permanent teeth (*X*_1_…*X*_7_). A linear regression model was created by choosing those variables that greatly contributed to chronological age using the stepwise selection method to get an estimate of age as a function of the morphological variables. The same sample was used to verify the constructed Egyptian-specific formula. Paired samples *t*-test was applied to assess the significances of the difference between DA and CA for the two formulas.

Ethical approval for this study was obtained from the ethical committee (REC), Faculty of Dentistry, Tanta University, and all methods were performed in accordance with its relevant guidelines and regulations.

## Results

There were no statistically significant intra-observer differences between the paired sets of measurements taken on the re-examined panoramic radiographs. Kappa values for all measurements were above 0.85, indicating a high intra-examiner consistency. A total of 1093 OPGs were collected which were assessed for the inclusion criteria. The study sample consisted of 762 OPGs which met the inclusion criteria. It included 354 girls (46.5%) and 408 boys (53.5%) with a mean chronological age of 9.92 ± 2.77 and 9.67 ± 2.5 years, respectively.

### Accuracy of Cameriere’s original formula

The mean DA of females, using Cameriere et al.’s original formula, was 9.34 ± 2.39 years with an underestimation of − 0.59 ± 0.96 years while the mean DA age of boys was 9.14 ± 2.13 years with an underestimation of − 0.53 ± 0.97 years (*p* < 0.001) (Table 2). In all female age groups, there was a significant difference between CA and DA except for 5 < 7 age group in which CA was overestimated (0.26 ± 0.71 years). On the other hand, in all male age groups, there was a significant difference between CA and DA except for 9 < 11 age group.

**Table 2:** The mean CA and mean estimated DA using Cameriere et al.’s original formula and the difference between DA and CA for both sexes and all age groups

Age group	Male						Female					
	*n*	Mean ± SD of age in years			*t*	*p*	*n*	Mean ± SD of age in years			*t*	*p*
		CA	Cameriere formula DA	Cameriere formula DA-CA (95% CI)				CA	Cameriere formula DA	Cameriere formula DA-CA (95% CI)		
5	51	5.94 ± 0.43	6.46 ± 0.70	0.52 ± 0.66 (0.33–0.71)	5.606	< 0.001	72	5.99 ± 0.65	6.25 ± 0.84	0.26 ± 0.71 (0.10–0.43)	3.131	0.003
7	103	7.99 ± 0.54	7.47 ± 0.97	− 0.52 ± 0.87 (− 0.69–(− 0.35))	6.101	< 0.001	92	7.93 ± 0.56	7.46 ± 0.97	− 0.47 ± 0.84 (− 0.65–(− 0.30))	5.363	< 0.001
9	84	10.01 ± 0.58	9.77 ± 0.92	− 0.23 ± 0.83 (− 0.42– (0.05))	2.591	0.011	95	10.12 ± 0.65	9.63 ± 0.92	− 0.49 ± 0.83 (− 0.66–(− 0.32))	5.762	< 0.001
11	74	11.80 ± 0.58	10.78 ± 0.76	− 1.01 ± 0.63 (− 1.16–(− 0.87))	13.892	< 0.001	86	12.08 ± 0.51	11.15 ± 0.90	− 0.93 ± 0.89 (− 1.12–(− 0.74))	9.674	< 0.001
13–15	42	13.91 ± 0.71	12.33 ± 1.22	− 1.58 ± 0.65 (− 1.78–(− 1.37))	15.696	< 0.001	63	14.09 ± 0.61	12.69 ± 0.78	− 1.40 ± 0.79 (− 1.60–(− 1.20))	14.025	< 0.001
All	354	9.67 ± 2.50	9.14 ± 2.13	− 0.53 ± 0.97 (− 0.63–(− 0.43))	10.365	< 0.001	408	9.92 ± 2.77	9.34 ± 2.39	− 0.59 ± 0.96 (− 0.68–(− 0.49))	12.289	< 0.001

### Establishment of new Egyptian formula

After testing the original formula of Cameriere et al.’s on the Egyptian sample and the resulted underestimation of dental age, a new specific formula was executed to get more precise results. The regression analysis revealed that not all the variables used in the cameriere original equation were significant predictors of age in the Egyptian sample. The results of the regression analysis showed that the normalized measurement of left mandibular first premolar (***X***_**4**_), the normalized measurement of left mandibular second molar (***X***_**7**_) and ***N***_**0**_ contributed significantly to the CA (Tables 3 and 4). The regression model yielded the following formula:

**Table 3:** Linear regression analysis for predictors of chronological age

Variables	Value	Standard error	*T*	*P*
Constant	9.943	0.196	50.853	< 0.001
*X*_1_	0.192	0.832	0.231	0.817
*X*_2_	0.668	0.867	0.770	0.441
*X*_3_	− 0.673	0.485	1.387	0.166
*X*_4_	− 1.738	0.510	3.409	0.001
*X*_5_	− 0.002	0.339	0.005	0.996
*X*_6_	0.648	0.666	0.973	0.331
*X*_7_	− 1.533	0.205	7.487	< 0.001
*N*_0_	0.637	0.039	16.160	< 0.001

**Table 4:** Linear regression of significant predictors

Variables	Value	Standard error	*T*	*p*
Constant	9.766	0.170		
*X*_4_	− 1.831	0.365	5.018	< 0.001
*X*_7_	− 1.393	0.151	9.238	< 0.001
*N*_0_	0.662	0.037	17.957	< 0.001

DA = 9.766 − 1.831 *X*_4_ − 1.393 *X*_7_ + 0.662 *N*_0_

*R*^*2*^ = 0.854, Adjusted *R*^*2*^ = 0.853

2 $$\mathrm{DA}=9.766-1.831 {X}_{4}-1.393 {X}_{7}+0.662 {N}_{0}\left({R}^{2}=0.854, \mathrm{adjusted }{R}^{2}=0.853\right)$$

**Fig. 3:**
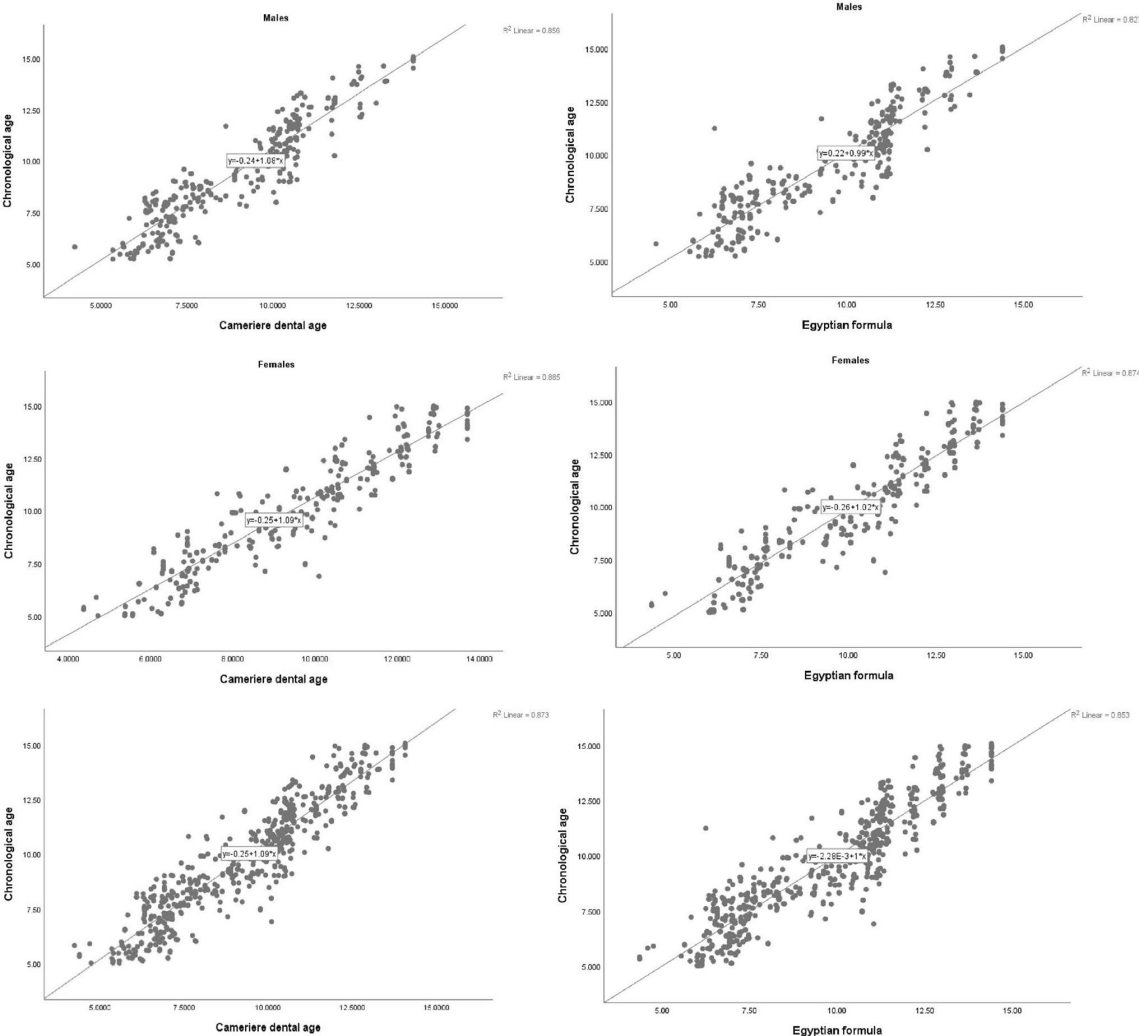
Plots of the chronological against estimated age in males and females for both equations

The regression model fits reasonably well with the trend as presented in Fig. 3 which shows a plot of CA versus DA for both equations.

### Accuracy of the new Egyptian formula

To prove that the Egyptian formula is more accurate than the Cameriere’s original formula, it was tested on the same sample. It underestimated the males age by − 0.12 ± 1.04 years and overestimates the females age by 0.1 ± 0.98 years (Table 5) with no statistically significant difference (*p* > 0.05). The predicted age in both girls and boys was slightly overestimated in younger children and underestimated in older ones, with a highly statistically significant difference (*p* < 0.001). Females, on the other hand, had more accurate results than males.

**Table 5:** The mean CA and mean estimated DA using the Egyptian formula and the difference between DA and CA for both sexes and all age groups

Age group	Male						Female					
	*n*	Mean ± SD of age in years			*t*	*p*	*n*	Mean ± SD of age in years			*t*	*p*
		CA	Egyptian formula DA	Egyptian formula DA-CA (95% CI)				CA	Egyptian formula DA	Egyptian formula DA-CA (95% CI)		
5	51	5.94 + 0.43	6.63 ± 0.65	0.69 ± 0.63 (0.51–0.87)	7.739	< 0.001	72	5.99 ± 0.65	6.69 ± 0.85	0.70 ± 0.82 (0.51–0.89)	7.262	< 0.001
7	103	7.99 + 0.54	7.76 ± 1.19	− 0.24 ± 1.08 (− 0.45–(− 0.03))	2.256	0.026	92	7.93 ± 0.56	7.99 ± 1.18	0.05 ± 1.03 (− 0.16 to 0.27)	0.495	0.622
9	84	10.01 + 0.58	10.38 ± 1.06	0.37 ± 0.95 (0.16–0.58)	3.566	0.001	95	10.12 ± 0.65	10.49 ± 0.99	0.37 ± 0.92 (0.19–0.56)	3.967	< 0.001
11	74	11.80 + 0.58	11.30 ± 0.93	− 0.50 ± 0.80 (− 0.68–(− 0.31))	5.343	< 0.001	86	12.08 ± 0.51	11.97 ± 0.88	− 0.11 ± 0.87 (− 0.29 to 0.08)	1.138	0.258
13–15	42	13.91 + 0.71	12.78 ± 1.14	− 1.13 ± 0.59 (− 1.31–(− 0.94))	12.341	< 0.001	63	14.09 ± 0.61	13.46 ± 0.74	− 0.63 ± 0.75 (− 0.82–(− 0.44))	6.640	< 0.001
All	354	9.67 + 2.50	9.55 ± 2.30	− 0.12 ± 1.04 (− 0.23–(− 0.01))	2.180	0.030	408	9.92 ± 2.77	10.03 ± 0.26	0.10 ± 0.98 (0.01–0.20)	2.120	0.035

## Discussion

Dental maturity estimation gives valuable information for precise diagnosis and treatment planning especially for pedodontists and orthodontists. It is critical for detecting delayed or advanced maturation also, understanding permanent tooth growth [45]. Thus, dental maturity estimation must be performed as accurately and precisely as possible. Healthy children of age group 5–15 years were included in the study because this age range is widely accepted for estimating a child’s dental age since teeth development passes through several stages during this period. The OPGs that displayed missing permanent tooth buds in the left mandibular quadrant were omitted because the existence of all lower left seven permanent teeth is required for proper estimations in Cameriere’s original method [46]. OPG was used in the present study because intraoral radiography is difficult to obtain in children without image distortion [47]. It provides a comprehensive visualization of the whole dentition; also, it minimizes children’s radiation exposure when compared to taking a radiographic survey of the full mouth [48].

Numerous morphological methods for estimating dental age have been established in which their accuracy is identified by the ability to arrive at an age as close as possible to the chronological age with acceptable error limits [49, 50]. Cameriere et al. [27] formulated a non-destructive method for age estimation in the Italian children, which was selected in the current study because it uses proportions (apex aperture by tooth length) instead of absolute numbers to eliminate any distortions caused by magnification and angulation issues [51]. It should be noted that different dental age estimation methods did not deliver a popular formula for the entire world’s population [52]. These methods also differed in their accuracy when different populations were examined. Hence this paper focused on improvement of the Cameriere et al., [27] method to suit the Egyptian children.

The applicability of Cameriere et al.’s original formula was tested in the present study before adapting it. Our results showed that Cameriere method yielded a mean underestimation of − 0.53 and − 0.59 years for boys and girls respectively; this is in accordance with Shrestha et al. [53] who found underestimation of the dental age by − 0.11 and − 0.23 years for Indian boys and girls respectively and Gulashi et al. [54] who reported underestimation of the dental age by − 0.47 and − 0.24 years for Turkish boys and girls respectively. Moreover, Elbakary et al.’s [43] study on Egyptian children revealed that Cameriere’s original formula resulted in an average underestimation of − 0.26 years for girls and − 0.49 years for boys, which is consistent with the present study results. On the other hand, these results disagreed with De Luca et al., [55] who reported that Cameriere method accurately estimated the age with age difference of 0.00 in Mexican boy sample and overestimated the dental age by 0.1 year in girls; the great influence of immigrant people of European origin mostly from Italy to Mexico City probably explains this high correlation. The maximum DA–CA difference using Cameriere et al.’s original formula was − 1.58 years at 13–15 years age group in males, while in females, the maximum DA–CA difference was − 1.40 years at the same age group; this high underestimation of older ages was agreed with Guo et al.’s [37] and Frucht et al.’s [56] study results.

In this study, an Egyptian-specific formula was fitted on the sample of 762 child, and it was found that the 1^st^ premolar and 2^nd^ molar variables had a significant correlation with age estimation and therefore were included in the adapted regression equation. These teeth, in our opinion, are acceptable for age estimation for many reasons. The second molar is the last tooth to complete its development, between the ages of 14 and 15 years, so it could be an effective variable for assessing age up to this age. Also, its crown stays intact for a long time because it is formed between the ages of 7 and 8 [57] and its eruption is predicted between the ages of 11 and 13. Furthermore, it is less prone to attrition than other teeth that mature earlier in life, so there is no reduction in clinical crown length, which could affect tooth ratio and final age estimation. Moreover, the mandibular first premolar has a low incidence to be congenitally missed [58]; also, it has acceptable tooth position for radiological analysis. This result disagreed with Cameriere et al.’ s original formula in which the developmental stage of 2^nd^ premolar contributed significantly to the regression formula. In a study from North China [37], the canine, 1^st^ premolar, and 2^nd^ molar were significant predictors of age estimation. Also, in an Indian study conducted by Attiguppe et al. [59], the 1^st^ premolar was included in the regression equation as a significant contributor. In the Serbian population [42], it was found that the canine and 2^nd^ molar were the most significant variables. Moreover, in North German children, Halilah et al. [41] adapted the Cameriere et al.’s European formula and found that the canine contributed significantly to his specific regression formula. It is worth noting that none of the teeth, nor sex, played a significant role in the Indian formula developed by Rai et al. [36].

When the Egyptian-specific formula was tested on each female age group separately, it was more accurate than the Cameriere’s original formula in most age groups. However, Cameriere et al.’s original formula yielded slightly better results at age group 5 with an overestimation of 0.26 years in comparison to an overestimation of 0.7 years at the same age using the Egyptian formula. On the other hand, the Egyptian formula in males resulted in accurate DA estimation at age groups 7, 11, and 13 with mean differences of − 0.24, − 0.5, and − 1.13 years, respectively. The Cameriere’s original formula, however, revealed more accurate results on males at the age of 5 and 9 with a DA–CA being 0.52 years and − 0.23 years in comparison to 0.69 years and 0.37 years using the Egyptian formula. In relation to the total sample, the Egyptian formula underestimated the dental age by − 0.0008 year; this agreed with Fernandes et al. [51], Shrestha et al. [53], and Gulashi et al. [54] who found an average underestimation of the age by − 0.04 year in Brazilian children, − 0.18 year in Indian children, and − 0.35 years in Turkish children respectively. When Egyptian formula was applied to older children (13–15 years age group), for boys, the mean DA was underestimated by − 1.13 years while for girls, it was underestimated by − 0.63 years; this agreed with Latić-Dautović et al. [60] who found the greatest underestimation for the last 14-year-old group in Bosnia and Herzegovina children. This significant decrease in accuracy in this age group can be attributed to the almost complete maturation of the teeth.

This is the first research to adapt Cameriere et al.’s original formula on an Egyptian sample to develop an Egyptian-specific prediction formula and to compare its accuracy for age estimation with the previous original formula. Different factors such as socioeconomic status, nutrition, dietary habits, lifestyle, and genetics can influence children’s growth in a country as large as Egypt. Future research should investigate the effects of the sample’s regional background, gender, nutrition, and chronological age distribution on the accuracy and reliability of dental age assessment in Egyptian children, moreover, to evaluate the applicability of this specific formula on another sample size and to compare its reliability with other methods of age estimation.

## Conclusion

It can be concluded from the study results that there is significant correlation between age and measurement of open apices of teeth. Also, it can be confirmed that the new specific formula was fairly accurate compared to Cameriere’s original formula. It has better accuracy for age estimation in Egyptian boys than girls. This accuracy decreases simultaneously with the completion of a child’s dental development.

## Data Availability

The datasets generated and analysed during the current study are not publicly available due to their containing information that could compromise the privacy of research participants but are available from the corresponding author on reasonable request.
